# Updated distribution of the invasive *Megachile sculpturalis* (Hymenoptera: Megachilidae) in Italy and its first record on a Mediterranean island

**DOI:** 10.3897/BDJ.8.e57783

**Published:** 2020-11-26

**Authors:** Enrico Ruzzier, Mattia Menchetti, Laura Bortolotti, Marco Selis, Elisa Monterastelli, Leonardo Forbicioni

**Affiliations:** 1 Universtità degli Studi di Padova, Legnaro (Padova), Italy Universtità degli Studi di Padova Legnaro (Padova) Italy; 2 World Biodiversity Association onlus c/o NAT LAB Forte Inglese, Portoferraio (LI), Italy World Biodiversity Association onlus c/o NAT LAB Forte Inglese Portoferraio (LI) Italy; 3 Università degli Studi di Firenze, Firenze, Italy Università degli Studi di Firenze Firenze Italy; 4 Council for Agricultural Research and Economics, Research Centre for Agriculture and Environment, Bologna, Italy Council for Agricultural Research and Economics, Research Centre for Agriculture and Environment Bologna Italy; 5 Via dei Tarquini 22, Viterbo, Italy Via dei Tarquini 22 Viterbo Italy

**Keywords:** biodiversity loss, early detection, invasive species, island, native plants, pollinators

## Abstract

*Megachile sculpturalis* (Smith, 1853) (Hymenoptera: Megachilidae) is an invasive solitary bee that is rapidly spreading all over Europe. The present study aims to update the distribution of this species in Italy. The research led to the collection of 177 records, obtained through bibliographic research and data-mining from websites, blogs and social networks. We here present the first record of *M. sculpturalis* on a Mediterranean island and discuss its possible effect on the native ecosystem. Given the particular discovery of *M. sculpturalis* on Elba Island (Tuscany), we suggest possible monitoring, containment and possible eradication measures of the species.

## Introduction

Invasive alien species are a threat to native biodiversity (Pyšek and Richardson 2010, Early et al. 2016) and are directly involved in ecosystem degradation, especially in coastal areas, inland waters, islands and Mediterranean-climate zones (Hulme et al. 2008, Gaertner et al. 2009, Russel et al. 2017). The Mediterranean basin is one of the biodiversity hotspots most at risk from terrestrial invasive species due to its central role in the world trade and the high human population density (Medail and Quezel 1999, Hopkins 2002). Invasive and exotic Apoidea constitute a serious menace to native bees due to disturbance, transmission of parasites and pathogens and competition for trophic resources and nesting sites (Goulson 2003, Traveset and Richardson 2006, Stout and Morales 2009, Russo 2016). However, knowledge relative to the effects caused by exotic on native bees, solitary in particular, is still scarce and fragmented (Bosch 1992, Gibbs and Sheffield 2009, McKinney and Park 2012, Graham et al. 2018), especially in islands (Rasmussen et al. 2012, Groom et al. 2014). Furthermore, invasive bees could represent a serious threat to ecosystems due to their disruption of local plant-pollinator interactions (Traveset and Richardson 2006, Aizen et al. 2008, Stout and Morales 2009) and invasive plants mutualism (Abe et al. 2010).

*Megachile* Latreille, 1802 (Megachilidae: Megachilini) is a rather specious genus of solitary bees with introduced species in almost all continents (e.g. Pitts-Singer and Cane 2011, Sheffield et al. 2010, Strange et al. 2011, Rasmussen et al. 2012, Russo 2016, Bortolotti et al. 2018, Gonzalez et al. 2019). Megachile (Callomegachile) sculpturalis (Smith, 1853) is a large-sized bee (18-39 mm in length) with an opportunistic nesting behaviour (Michener 2007): it uses pre-existing nests of other bees (i.e. Carpenter Bees) or compete for pre-existing cavities with other cavity nesting species (i.e. Leaf Cutter Bees) (Laport and Minckley 2012, Roulston and Malfi 2012, Le Féon et al. 2018), beetle galleries (Kovács 2015) or artificial structures, such as brick holes, plastic tubes (Zandigiacomo and Grion 2017, Aguado et al. 2018) and bee hotels (Gihr and Westrich 2013, Quaranta et al. 2014, Dillier 2016, Geslin et al. 2020). Brood cells and nest closures are created using wood fibres, leaf fragments, clay and resin (Michener 2007). The species is polylectic for nectar at adult stage, feeding on a wide range of flowering plants (Suppl. material 1), while it shows a marked selection for pollen to use as a food source for larvae (Quaranta et al. 2014, Andrieu-Ponel et al. 2018, Le Féon and Geslin 2018). *Megachile sculpturalis*, native to the Eastern Palaearctic (Korea, Japan, China, Taiwan), is widely recognised as invasive for its great capability to establish in a wide range of environments outside of its native geographical habitat (Le Féon and Geslin 2018). This species was first recorded outside of its range in North Carolina in 1994 (Mangum and Brooks 1997) and is now distributed from northern Mexico to Canada (Magnum and Bambara 1998, Kondo et al. 2000, Mangum and Sumner 2003, Paiero and Buck 2003, Hinojosa-Diaz et al. 2005, Hinojosa-Díaz 2008, O’Brien and Craves 2008, Mazurkiewiez 2010, Dellinger and Day 2014, Parys et al. 2015, Campbell et al. 2016). *Megachile sculpturalis* was intercepted in France in 2008 (Vereecken and Barbier 2009) and it rapidly spread over Europe: Italy in 2009 (Quaranta et al. 2014), Switzerland in 2010 (Amiet 2012), Hungary in 2015 (Kovács 2015), Germany in 2015 (Westrich et al. 2015), Austria in 2017 (Le Féon et al. 2018), Slovenia in 2016 (Gogala and Zadravec 2018), Spain in 2018 (Aguado et al. 2018, Ortiz-Sánchez et al. 2018), Ukraine in 2019 (as “Crimea”, Ivanov and Fateryga 2019) and Liechtenstein (Lanner et al. 2020). Specifically referring to Italy, published records of *M. sculpturalis* are scattered (Quaranta et al. 2014, Zandigiacomo and Grion 2017, Grossi et al. 2018 Moldoveanu 2019, Poggi et al. 2020).

Since no exhaustive reference exists about the real extent of the invasion of *M. sculpturalis* in the Italian peninsula, we decided to conduct extensive research in order to fill the knowledge gap and to update the distribution of this invasive species. In particular, we give emphasis to the discovery of *M. sculpturalis* on Elba Island (Tuscan Archipelago), the first case of an exotic bee on a Mediterranean island and discuss the possible effects on the native flora and fauna. Given the uniqueness of the discovery, we suggest how Elba Island and the Tuscan Archipelago National Park may become a model for monitoring, controlling and even eradicating this invasive bee in island ecosystems.

## Materials and methods

### Data collection

In order to have the most efficient and extensive data collection, we adopted a mixed data search approach: literature review, direct observation, data-mining and dedicated websites. Direct observations were recorded through active research by the authors or via communication with other entomologists. Further data were mined from national entomological and naturalists’ online forums (“Forum Entomologi Italiani”, “Forum Natura Mediterraneo”), Facebook groups (“Entomologia, Insetti e altri Artropodi”, “Insetti e Aracnidi Italiani”) and national and international citizens' science websites (iNaturalist.org, Beewatching.it, Stopvelutina.it). All the data collected are updated to December 2019. In the final database, we included only records verified by the authors through pictures, in possession of the precise location and other relevant information. For each observation, we recorded the date, locality [name], GPS coordinates (if available), number of observed specimens (if defined), landscape context, data source, nesting observations (bee hotels or natural nests) and flower interactions. Since it was not always possible to identify the sex of the specimens, the sex category was not included in the analysis.

All the data collected are available in Suppl. material 2.

### Distribution map

The maps have been made with QGIS (v. 3.4.2-Madeira) using a raster layer freely available on Natural Earth (www.naturalearthdata.com) and later edited with Adobe Illustrator CC 2019.

### DNA barcoding

A tissue sample of one of the specimens collected on Elba Island was sent to and sequenced at the Canadian Centre for DNA Barcoding (CCDB, Biodiversity Institute of Ontario, University of Guelph). DNA sequencing resulted in a COI barcode fragment of 658 bp. The sequence, named MOLTE082-19, is privately stored as part of the project “MOLTE” in the Barcode of Life Data Systems (BOLD; Ratnasingham and Hebert 2007). The integrated bioinformatics platform BOLD was used to assess the identity of the sequence obtained. Furthermore, the sequence was compared to six *M. sculpturalis* sequences available in BOLD, namely ABBOL043-15; BCT012-06; BEECA275-06; BEECA276-06; GBMIN78089-17 and MOLTE077-19. These barcodes represent processed IDs in BOLD and are grouped under the Barcode Index Number (BIN) BOLD:AAE8645. The pairwise genetic distances between sequences were calculated using MEGA X software, under default settings (Kumar et al. 2018). The haplotype network was built with the programme TCS 1.21 (Clement et al. 2000) and later edited with tcsBU (Múrias dos Santos et al. 2015) and Adobe Illustrator CC 2019.

## Results

The survey produced 177 records covering most of the Italian peninsula. Northern regions present the highest percentage of observations (80.2% of the total), respectively: 33 (Lombardy), 31 (Emilia-Romagna), 22 (Veneto), 21 (Liguria), 16 (Piedmont), 12 (Trentino-Alto Adige) and 7 (Friuli-Venezia Giulia). Central Italy proved to be just as colonised although, to a lesser extent (18.1%): 22 (Tuscany), 5 (Lazio), 3 (Abruzzo), 1 (Marche) and 1 (Umbria) while three regions in the South (Campania, Calabria and Basilicata) possess one record each (1.7%). So far, the species is not yet recorded in Valle d’Aosta, Molise, Sicily and Sardinia. Data show that the number of reports increased exponentially from the sporadic reports per year between 2009 and 2015, to 11, 21, 39, 97 reports in 2016, 2017, 2018 and 2019, respectively.

Analysing the sources of our data, Facebook results in the primary source of records (49, about the 27.7% of the total), followed by direct observations (41, 23%), iNaturalist (39, 22%), Beewatching (13, 7.3%), literature review (17, 9.6%), online forums (10, 5.6%) and Stopvelutina website (8, 4.5%) .

The altitudes at which *M. sculpturalis* was recorded range from the sea level (~ 1 m) up to slightly above 1400 m (max. 1421 m a.s.l.), with an average of 212.3 m. Most of the reports come from plain areas, 68 of which were below 50 m a.s.l. (38.4%) and 62 comprised between 50 and 300 m a.s.l. (35%); 33 reports come from hilly areas between 300 and 700 m a.s.l. (18.6%) and 12 from mountain locations above 700 m a.s.l. (6.8%), five of which are above 1000 m.

Observations present a peak of detections in July (113, 63.8%) followed by August (49, 27.7%), June (13, 7.3%) and September (2, 1.1%).

Regarding the landscape, most of the observations were made in rural areas (91, 51.4%), followed by the urban (69, 38.9%) and natural environment (17, 9.6%).

In 68 cases out of 177 (38.4%), the foraging behaviour of *M. sculpturalis* was observed and the visited plant were recorded. *Lavandula* sp. L. (Lamiaceae) is the most visited plant with 26 observations (38.2%), followed by *Wisteria* spp. (*Wisteria* sp. Nutt. + *Wisteria sinensis* (Sims) DC + *Wisteria floribunda* (Willd.) DC.) (Fabaceae) with 10 cases (14.7%). The other observations identified: *Syringa* sp. L. (Oleaceae) (4), *Vitex agnus-castus* L. (Lamiaceae) (4), *Chamaerion angustifolium* (L.) Scop. (Onagraceae) (3), *Cirsium* sp. Mill. (Asteraceae) (3), *Citrus* spp. (*Citrus* sp. L. + *Citrus japonica* Thunb.) (Rutaceae) (2), *Styphnolobium japonicum* (L.) Schott (Fabaceae) (2), *Cichorium intybus* L. (Asteraceae) (1), *Cirsium arvense* (L.) Scop. (Asteraceae) (1), *Firmiana platanifolia* (L.) W. Wight (Malvaceae) (1), *Helianthus annuus* L. (Asteraceae) (1), *Koelreuteria paniculata* Laxm. (Sapindaceae) (1), *Lavandula angustifolia* Mill. (Lamiaceae)(1), *Ligustrum lucidum* W.T. Aiton (Oleaceae) (1), *Rubus* sp. L. (Rosaceae) (1), *Scabiosa* sp. L. (Caprifoliaceae) (1), *Tetradium daniellii* (Benn.) T.G. Hartley (Rutaceae) (1), *Trifolium pratense* L. (Fabaceae) (1) and unidentified plants (3). A total of 37 nesting reports were done in bee hotels (20.9%), with 21 in natural cavities (11.8%).

Literature review and our data allowed the identification of the most commonly visited genera of plants all over the invaded range:

*Styphnolobium* spp. Schott (Fabaceae) (Mangum and Sumner 2003, Hinojosa-Diaz et al. 2005, Matteson et al. 2008, Laport and Minckley 2012, Gihr and Westrich 2013, Westrich et al. 2015, Aguado et al. 2018, Andrieu-Ponel et al. 2018, Andrieu-Ponel et al. 2018, Le Féon et al. 2018); *Ligustrum* spp. L. (Oleaceae) (Batra 1998, Mangum and Sumner 2003, Hinojosa-Diaz et al. 2005, Norden 2008, Laport and Minckley 2012, Quaranta et al. 2014, Aguado et al. 2018, Andrieu-Ponel et al. 2018, Zandigiacomo and Grion 2017), *Pueraria* spp. A. DC. (Fabaceae) (Iwata 1933, Mangum and Brooks 1997, Batra 1998, Laport and Minckley 2012, Quaranta et al. 2014, Buchmann and Ascher 2015) and *Lathyrus* spp. Mill. (Fabaceae) (Ascher 2001, Mangum and Sumner 2003, Hinojosa-Diaz et al. 2005, O’Brien and Craves 2008, Dillier 2016). As a result of our survey, *Chamaerion angustifolium*, *Helianthus annuus*, *Syringa* sp. *and Tetradium daniellii* are documented here for the first time as feeding plants.

## Discussion

**Figure 1.**
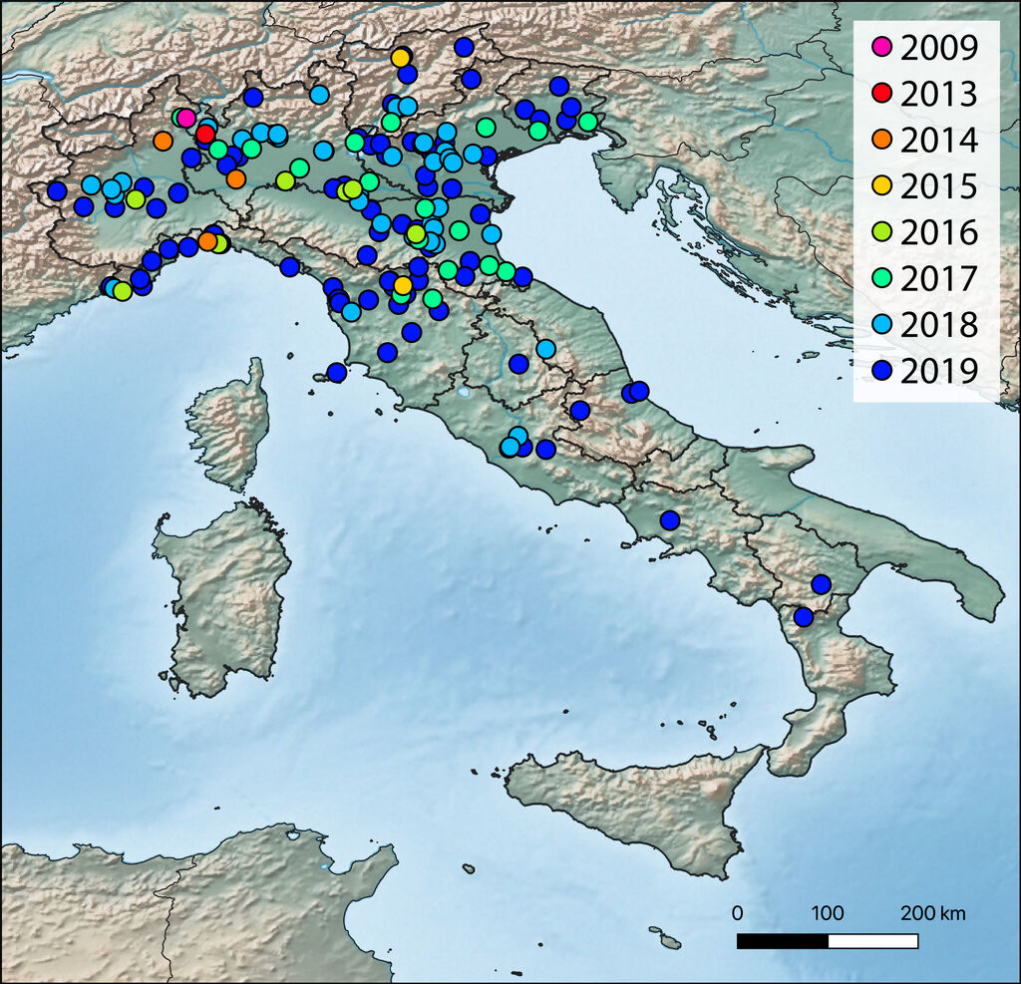
Distribution map of Megachile (C.) sculpturalis in Italy: records subdivided per year

**Figure 2.**
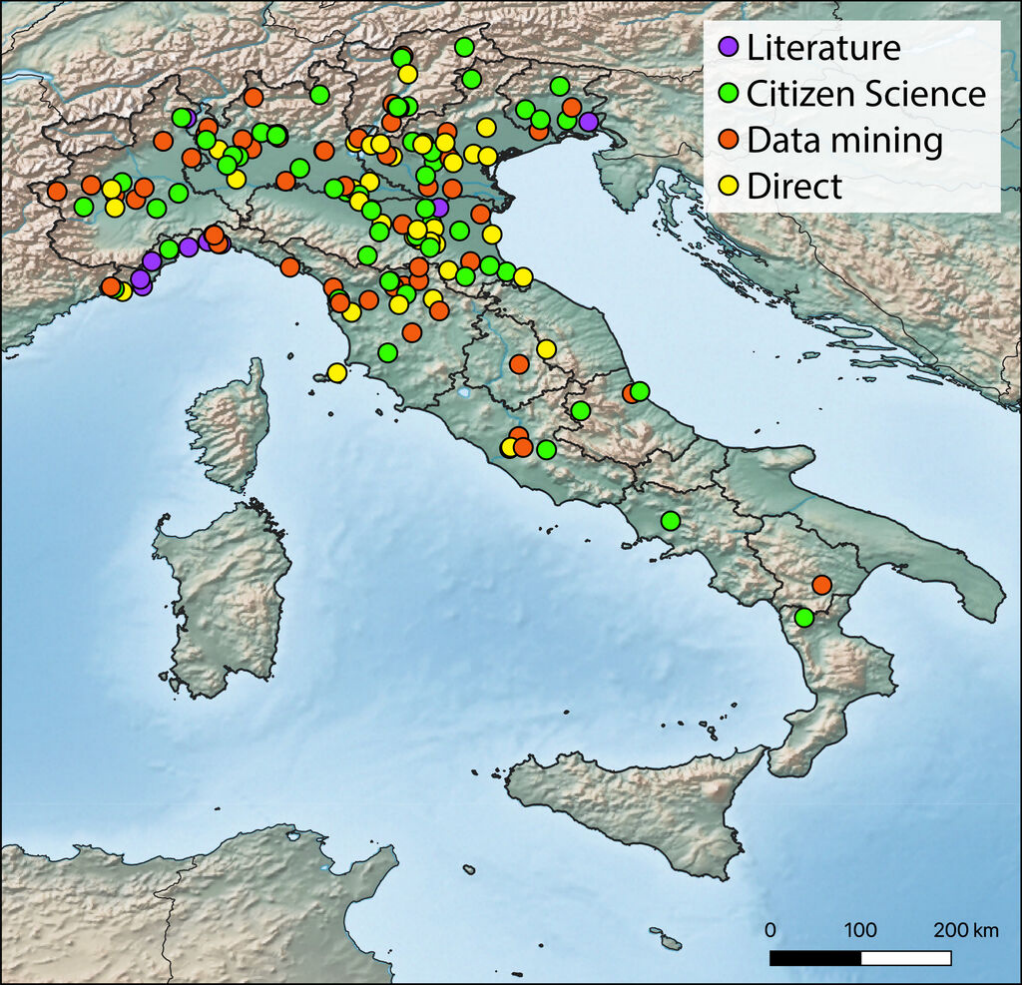
Distribution map of Megachile (C.) sculpturalis in Italy: records subdivided per data sources.

Since its first discovery in Italy in 2009 (Quaranta et al. 2014), *M. sculpturalis* has spread rapidly throughout the country, with the exception of Sardinia and Sicily, where apparently, it is not yet established (Fig. 1). The species seems to be fairly common and widely distributed in the central-northern part of the peninsula, while it is still sporadic and localised in the south. Its establishment success in northern Italy is probably attributable to its early arrival in the area, the important commercial traffic which facilitated its passive diffusion and the general state of environmental degradation that characterises the Po Valley and northern Italy in general. However, the scattered presence of *M. sculpturalis* in southern Italy could be the result of sampling bias rather than the relative rarity of this species in the area (Fig. 2); further investigations are needed to ascertain this condition. *Megachile sculpturalis* is a particularly plastic and adaptable species, capable of colonising environments from sea level up to over a thousand metres of altitude; however, as data suggest, it seems to have an optimum between one and three-hundred metres approximately. This preference is probably associable, in addition to the more favourable temperature, to the greater environmental degradation and degree of anthropisation of the territory; our data demonstrate a predominance of this species in disturbed ecosystems with about 85% of the observations documenting *M. sculpturalis* primarily in either urban or rural environments. This link between invasive species success and urbanisation has been recently proved by Fitch et al. (2019). The phenology of the Italian population of *M. sculpturalis* is limited to summer (late June - early September), with a peak of activity in July; it is interesting to note that this activity trend is similar to that observed in the rest of Europe (e.g. Aguado et al. 2018, Le Féon et al. 2018, Ortiz-Sánchez et al. 2018) and North America (e.g. Paiero and Buck 2003, O’Brien and Craves 2008). Due to the lack of knowledge on the phenology of *M. sculpturalis* in the countries of origin, it is not possible to develop any kind of comparison.

### The introduction into Elba Island (Tuscan Archipelago)

**Figure 3.**
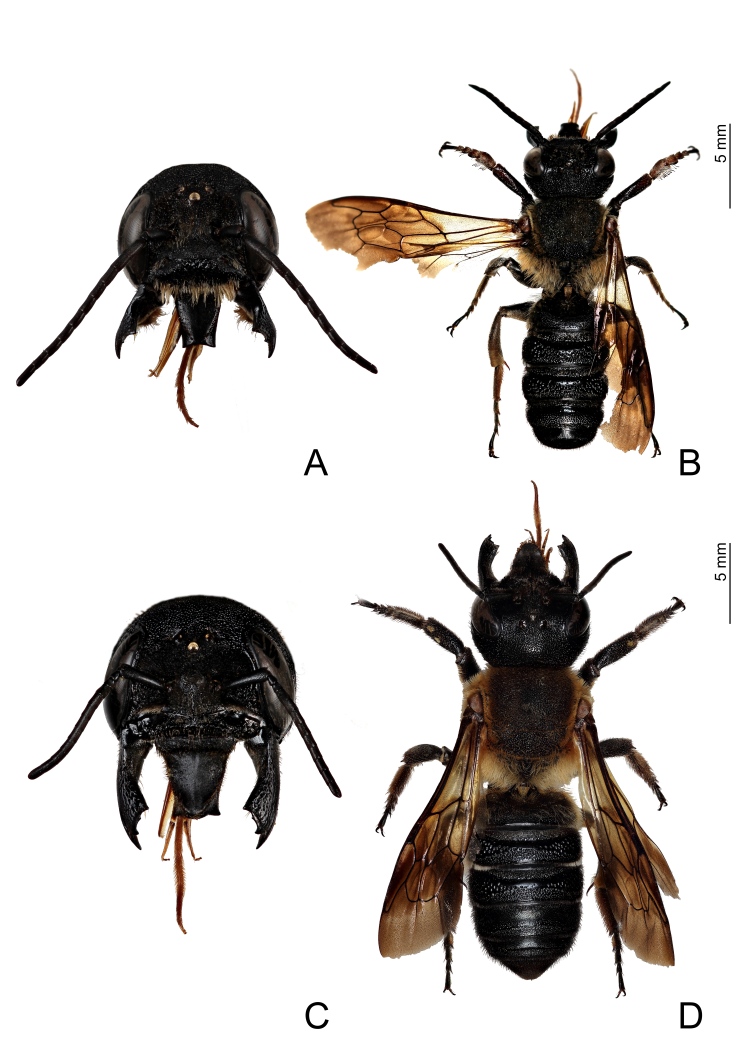
Two specimens of Megachile (Callomegachile) sculpturalis (Smith, 1853) collected on Elba Island. **A.** male, fontal view of the head; **B.** male, dorsal habitus; **C.** female, fontal view of the head; **D.** female, dorsal habitus.

The first specimen of *M. sculpturalis* (male) was collected in Rio, loc. Nisportino, on 16 August 2019, (42.832500, 10.386111) by Marco Selis. The author at the time of collection observed several specimens feeding on flowers of *Vitex agnus-castus* (Lamiaceae). On 17 August 2019, Leonardo Forbicioni collected three specimens (one male and two females) (Fig. 3) and had the opportunity to observe at least another ten, all in the same spot. On 5 September 2019, Enrico Ruzzier and Leonardo Forbicioni revisited the site to assess the presence of *M. sculpturalis*, but contrary to expectations, no specimens were observed.

**Figure 4.**
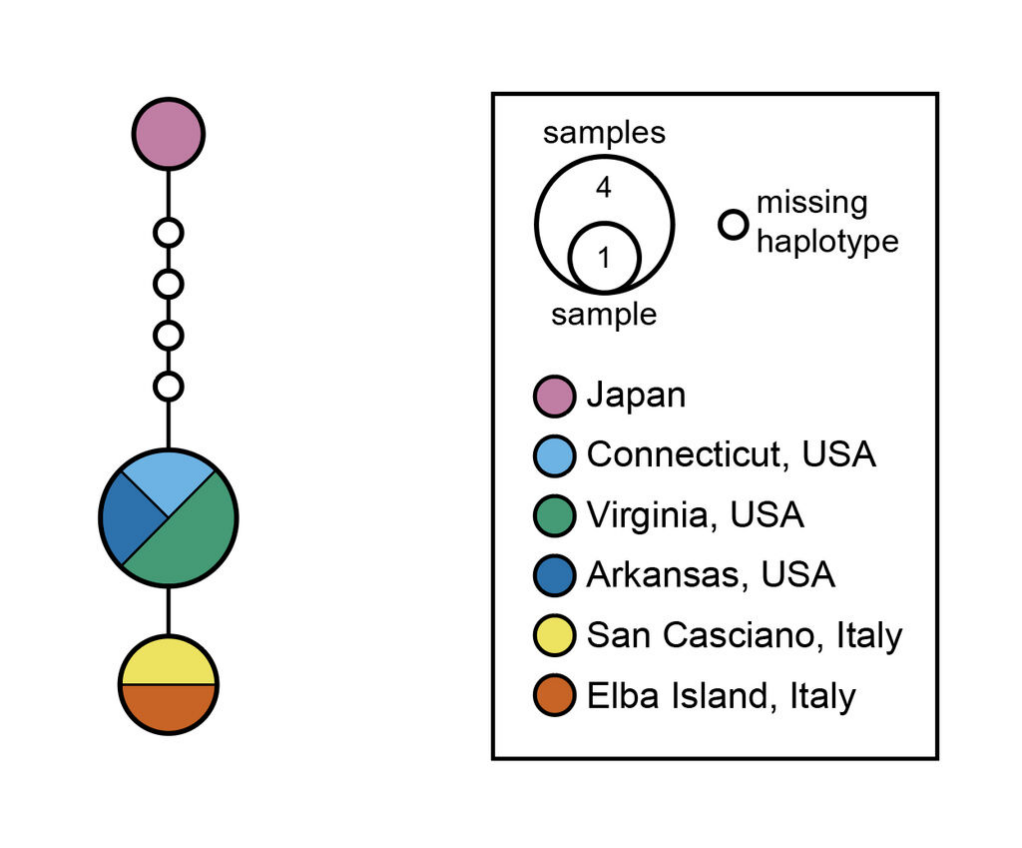
Haplotype network of mitochondrial COI sequences of Megachile (Callomegachile) sculpturalis (Smith, 1853).

Since Nisportino is a small village, geographically isolated from any important port and visited mainly by tourists, it is plausible that the introduction of *M. sculpturalis* could have occurred only locally and accidentally by transport of commodities; this hypothesis is suggested by the tendency of *M. sculpturalis* to nest, in absence of natural wood cavities, in plastic tubes, brick holes and other opportunistic shelters (Aguado et al. 2018). Given the abundant usage at the discovery site of imported bamboo for ornamental and construction purposes, it is more likely that this may be the introduction vector. The hollow bamboo is already recognised as a nesting site widely used by *M. sculpturalis* and other Megachilidae (Iwata 1933, Batra 1998), including in bee hotels (Quaranta et al. 2014, Dillier 2016, Geslin et al. 2020). The only means by which any goods are imported to Elba Island is direct naval transportation from the continent (Tuscany). We do not know if bamboo arrived at the cargo area already contaminated, but it is plausible that the material has been colonised during the period spent at the storage point at the port. A possible alternative for the introduction could be the importation of firewood from the Italian mainland; indeed, this represents a continuous and unregulated traffic. However, if the wood were actually the primary vector of introduction of *M. sculpturalis* on Elba Island, the distribution of the species would probably be associated with heavily populated areas rather than an isolated location, frequented almost exclusively during summer. COI identity between the Elban and Tuscan samples suggest a possible direct introduction from the continent. Due to an unsampled haplotype diversity in the native areas, the haplotype network analysis shows that the Elban and Tuscan sequences have a greater affinity to the American than to the Japanese one (Fig. 4).

In the near future, newly available DNA barcodes of *M. sculpturalis* from both the native and introduced areas will substantially contribute to clarify the relationships between the various populations and possibly highlight the main pathways of national and transnational introduction of the species. In particular, the complete haplotypes' characterisation of the population inhabiting the Italian peninsula may help to clarify if the current population is the result of multiple independent introduction events or if it is, instead, attributable to a single or a small group of founders. In addition to DNA barcodes, the usage of multiple and more informative genetic markers may constitute a powerful tool to reconstruct the invasion pattern of this species determining the population of origin and the source of introduction for all locations on a global scale.

Understanding this process could help the development of more effective control systems in limiting the spread of *M. sculpturalis* and, in particular, preventing its introduction into territories where it is still absent. With specific reference to our case, if not managed in an appropriate way, *M. sculpturalis* could spread amongst the islands of the archipelago and, in the same way, be introduced into Sardinia and Corsica.

### Possible effects on native flora

Invasive plants determine the loss of local biodiversity and modification of the landscape and their control and management imply important investments of both human and economic resources (IUCN 2008, Jardine and Sanchirico 2018). The Tuscan Archipelago currently counts thirty-one invasive plants of high management priority (Lazzaro et al. 2013, Ferretti et al. 2013, Lazzaro et al. 2014). The Arcipelago Toscano National Park (PNAT) has implemented and still develops management and eradication projects against some of these species, such as *Ailanthus altissima*, *Senecio angulatus*, *Carpobrotus* sp. and *Acacia saligna* (Zanichelli et al. 2014). Alien bees tend to be more efficient pollinators in comparison to oligolectic native bees (Traveset and Richardson 2006, Stout and Morales 2009) and may promote invasive mutualism in an insular pollination system. Abe et al. (2010) reported that reproduction of alien plants was facilitated by the flora preference of introduced bee species on islands. *Megachile sculpturalis* has shown a feeding preference for exotic plants originating from the same biogeographical context (Quaranta et al. 2014, Aguado et al. 2018, Le Féon and Geslin 2018) and some of these are already invasive in the archipelago. Therefore, the establishment of *M. sculpturalis* could favour, as well as accelerate, the spread of invasive plants, *Ailanthus* especially, thus determining the simplification of the environment and the drift towards a progressive replacement of the native flora. In particular, late summer-autumn blooming plants, bearing floral structures suitable for *M. sculpturalis*, may benefit most.

### Possible effects on native pollinators

The Apoidea of the Tuscan Archipelago are poorly studied (Generani et al. 2001, Anonymous 2014, Filippi and Strumia 2019, Forbicioni et al. 2019) and an overview regarding biology, community structures and pollination networks is still lacking. As a consequence, the effect of an exotic species on the local fauna is difficult to estimate. We maintain that the settlement and propagation of *M. sculpturalis* could have relevant repercussions similar to what was suggested by Reaser et al. (2007), Aizen et al. (2008), Kenis et al. (2008) and Stout and Morales (2009). Recently, Graham et al. (2018) demonstrated how the invasive *Anthidium manicatum* (Linnaeus,1758) (Megachilidae: Anthidiini) excludes the American native *Bombus impatiens* Cresson 1863 (Apidae: Bombini) from floral resources due to its aggressive and non-specific territorial behaviour. Results suggest that deprivation does not seem to significantly affect the growth and fitness of *B. impatiens* colonies. In the same contribution, the authors correctly argue that the apparent lack of effect of *A. manicatum* on *B. impatiens* is attributable to a compensatory action given by the colony and that the effects could be more significant in the case of solitary bees, whose nest construction depends on the activity of a single female (Graham et al. 2018). No aggressive behaviour has ever been observed in *M. sculpturalis* when feeding on flowers; however, it is plausible that its mere presence may constitute a source of disturbance for native bees, considerably reducing the time spent on foraging and forcing them to devote more attention to certain species of plants than others, as suggested in Stout and Morales (2009). A decrease in the quality and variety in the sources of nectar and pollen may have substantial repercussions on the fitness of local bees, solitary especially (Stout and Morales 2009, Graham et al. 2018). The loss of bee diversity could have important repercussions on the ecosystem service that this group offers, causing a general loss of pollination efficiency of the native flora. On the other hand, *M. sculpturalis* is a proven competitor for the reproduction sites of some Apoidea; in fact, its inability to build nests leads it to occupy Xylocopinae nests (i.e. *Xylocopa*) (Laport and Minckley 2012, Roulston and Malfi 2012) and to occupy cavities usually used by other large Megachilidae, such as *Anthidium* sp. Fabricius‎, 1805‎ and *Osmia* sp. Panzer‎, 1806‎ (Aguado et al. 2018, Le Féon et al. 2018). Despite Aguado et al. (2018) not reporting any aggression at the nest entrance, territorial behaviour at the nest proximity and directed against any other hymenopteran (regardless of species and size, E. Ruzzier *pers. obs.*) and the preliminary results published in Geslin et al. (2020) suggested a relevant displacing effect against those Apoidea sensitive to disturbance.

### Monitoring and control strategies

Considering the relatively small size of Elba Island and the only recent arrival of *M. sculpturalis*, the development of an efficient monitoring plan and effective control strategy is still potentially achievable; however, it is important to act promptly before the species can spread over the entire island and the whole archipelago. The monitoring plan, here proposed for Elba Island, can be equally used/repeated on the whole Italian territory, to homogenise the expansion information on *M. sculpturalis*. To develop an efficient monitoring and containment plan, four factors must be taken into consideration: ease of identification, nesting preferences, phenology and voltinism. Due to its large size and its characteristic appearance, *M. sculpturalis* can be easily recognised, even by less experienced citizens, as already proven during the data collecting presented in this paper and by Le Féon et al. (2018) and Lanner et al. 2020, while misidentification is sporadic. Adjoining the development of a scientific monitoring protocol, in this scenario, an extensive action developed with the contribution of Citizen Science is highly desirable, especially to maximise action efficiency by minimising the economic and resource investment. It should not be overlooked that Elba Island is part of the Tuscan Archipelago National Park and that there exists a close collaboration between the park and the local communities. The development of an adequate communication activity associated with specific seminars and training could potentially expand the pool of citizens capable of contributing substantially to the monitoring and control of this species. However, all activities must remain under the supervision of the Park Authority in order to avoid unconscious and autonomous actions that could cause damage to the environment. The data collected during our survey, combined with the information provided in literature, confirm the efficiency of bee hotels in detecting *M. sculpturalis*. Therefore, the use of bee hotels could constitute both a rapid and economic tool for spotting the species and an excellent medium for communicating the issue of invasive species and biodiversity conservation. It may also be considered that *M. sculpturalis* is tolerant in nest selection and may easily colonise bee hotels purposely positioned. Observations lead us to affirm that *M. sculpturalis* is present preferably in anthropised environments; consequently, bee hotels should be placed at the edge of the main towns or in the countryside, all areas easily accessible and therefore easier to monitor. The diameter of holes or canes used in bee hotels is the only condition that could substantially influence nest colonisation; for this reason, the internal diameter should range between 8.0 and 12.0 mm, as suggested by Aguado et al. (2018) and Geslin et al. (2020). Considering that *M. sculpturalis* is univoltine, the monitoring activities should be concentrated between late June and early September. Since the nests are completed and sealed starting from the middle of the summer, it is sufficient to remove them at the end of the reproductive season (autumn). Nest removal permits the destruction of the brood and the collection of important information, such as the abundance of the brood and types of pollen collected. We maintain that such control action perpetrated over time, thanks to the continuous subtraction of new generations, could help in controlling the growth of the population and potentially contribute to contain the diffusion of *M. sculpturalis*. To ensure the elimination of *M. sculpturalis* nests, it is necessary to mark them during the construction process; this activity, easy to carry out, allows the avoidance of unnecessary destruction of native bee nests. Furthermore, at the end of summer when the bee hotel is successfully colonised, it is possible to record new data about nesting density, displacing effect and other features related to the nesting behaviour of this species. This information may contribute to understanding the biology of *M. sculpturalis* and, where possible, to improve monitoring and the containment plans.

## Conclusions

The distribution of *M. sculpturalis* in the Italian peninsula from 2009 to date demonstrates the great spreading capacity of this species, which was able to colonise the whole country in a few years. An especial concern is given by its presence on Elba Island, which is part of Tuscan Archipelago National Park (PNAT) and represents the tourist and commercial route to Corsica and Sardinia. The particular conditions of vulnerability that occur on island ecosystems could expose them to concrete risks of alteration of their ecological balance by alien species. The observations, made in Europe and the USA, suggest a possible risk for the native flora and fauna due to *M. sculpturalis*, as a result of competition with local bees for foraging and nesting sites and for the spread of exotic plants. For this reason, we strongly suggest for this species a monitoring and containment action, which should also include an attempt to eradicate it from Elba Island. The results of the Italian monitoring highlight the relevance of Citizen Science contribution, made possible by the large size and the easy recognition of this species. The use of bee hotels, which represent an aggregation site for this species, could work favourably, both for the monitoring and for the control of the species, by destroying the nests at the end of the nesting season.

## Supplementary Material

59237F18-D052-5069-B1C5-AAAD1875A6A8 10.3897/BDJ.8.e57783.suppl1 Supplementary material 1 Megachile sculpturalis -feeding plants Data type biological data File: oo_443354.xlsx https://binary.pensoft.net/file/443354 Ruzzier, E., Monterastelli, E.

7CB78276-6DFC-504E-B449-868914EC5839 10.3897/BDJ.8.e57783.suppl2 Supplementary material 2 Cumulative dataset of the Italian localities of M. sculpturalis Data type distribution data File: oo_470389.xlsx https://binary.pensoft.net/file/470389 Bortolotti, L.. Monterastelli, E., Ruzzier, E.
